# Bilobed Flap Coverage for the Reconstruction of Dorsal Soft Tissue Defect Over the Proximal Phalanx: A Case Report

**DOI:** 10.7759/cureus.51538

**Published:** 2024-01-02

**Authors:** Fang Li, Min Kai Chang, Jinyuan Gan, Chung Ming Chan

**Affiliations:** 1 Department of Hand and Reconstructive Microsurgery, National University Hospital, Singapore, SGP; 2 Medical School, Duke-National University of Singapore (NUS) Graduate Medical School, Singapore, SGP

**Keywords:** orthopedic, dorsal, finger, bilobed flap, plastic surgery, hand surgery

## Abstract

Soft tissue defects over the dorsal finger are common and may result from trauma, burns, or surgical management of infections and tumors. We present a case where a bilobed flap was used for the reconstruction of a soft tissue defect dorsal to the proximal phalanx of the ring finger and discuss the design of this flap. The defect was secondary to a collar button abscess of the right third webspace and the surgical debridement required to control the infection. The exposed extensor tendon over the proximal phalanx required coverage. The bilobed flap was designed with the first lobe over the right middle finger proximal phalanx and the second lobe over the right second webspace and index finger. The flap healed uneventfully and the patient had good functional recovery. This design for a bilobed flap is suitable for soft tissue reconstruction of defects over the dorsum of the proximal phalanx. It is a simple, reliable, single-staged procedure that provides like-for-like reconstruction and has minimal donor site morbidity.

## Introduction

Soft tissue defects affecting the dorsum of fingers may result from a variety of conditions and their surgical management, including trauma, burns, soft tissue infections, and tumors. Surgical debridement of the dorsum of the hand and fingers can result in defects requiring secondary reconstruction, and the trauma and inflammation secondary to infection and prior surgery may compromise certain reconstructive options that depend on local perforators. While volar defects are challenging to cover with locoregional flap owing to the limited mobility of the palmar glabrous skin due to the fibrous septae that anchor the skin, reconstruction of the dorsal hand and fingers is challenging in its own right. There is a scarcity of available tissue with thin skin over the extensor apparatus and local flap options are similarly limited. Local flaps can be used for the dorsum of the hand offering a “like-for-like” cover. Local flap options include rotation flaps, reverse dorsal metacarpal artery flaps [1,2], and reverse cross-finger flaps [3,4]. Distant pedicled flaps (such as groin flaps) or free flaps can also be used for coverage of defects over the dorsum of fingers, but these are more complex and warranted in situations where locoregional flaps are unable to provide adequate coverage.

The bilobed flap was first described by Johannes Esser [5] in 1918 and is a random pattern local double transposition flap. It provides like-for-like reconstruction and recruits skin from sites with more tissue laxity. It is used extensively in the reconstruction of small-to-moderate-sized cutaneous facial defects [6] and, more recently, in lower limb defects [7,8]. In the hand, it has been used for coverage of small defects such as those resulting from digital mucous cyst excision [9], for the reconstruction of dorsal thumb defect following tumor excision [10], and for reconstruction of the webspace in syndactyly release [11]. In this case report, we present the use of a bilobed flap as a useful option for coverage of a dorsal finger defect over the proximal phalanx.

## Case presentation

**Figure 1:**
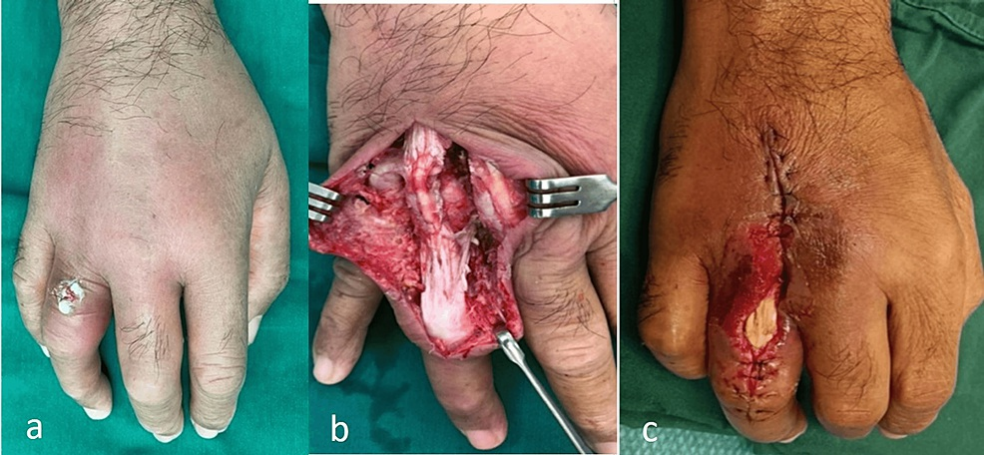
Appearance of the dorsal hand before flap surgery: (a) Appearance at presentation. (b) Appearance during debridement. (c) Defect following debridement.

A 60-year-old male developed a collar button abscess affecting his right third webspace with the abscess and associated cellulitis involving the dorsal soft tissue extensively. While he was diabetic, he did not recall any penetrating trauma. He underwent multiple surgical debridements from both volar and dorsal approaches. The volar wound was closed primarily; however, the dorsal approach for debridement resulted in a soft tissue defect over the dorsum of the proximal phalanx of the ring finger exposing the extensor tendon. The defect measured approximately 1.5 cm × 3 cm and could not be closed primarily (Figure 1).

**Figure 2:**
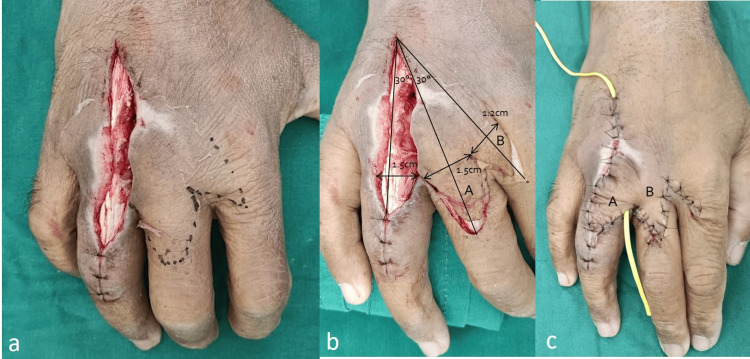
Flap design: (a) Design of the flap. (b) Considerations taken into planning the flap. (c) Transposed flap (yellow vessel loop placed as a drain removed postoperatively).

In planning for flap coverage of this defect, the options considered were a reverse cross-finger flap from the adjacent middle finger, a first dorsal metacarpal artery flap, a dorsal metacarpal artery perforator flap, as well as the bilobed flap described below. A reverse cross-finger flap would have required a second surgery for division of the flap as well as skin grafting over the transposed adipofascial flap. A first dorsal metacarpal artery flap was also considered; however, with the pivot point being at the proximal first webspace, it was uncertain if the flap would cover the most distal end of the defect and would have required skin grafting of the donor site defect. Lastly, while a dorsal metacarpal artery perforator flap was considered, it was uncertain if prior surgical debridement would have compromised the perforator. Hence, a bilobed flap was chosen to achieve closure of this wound owing to its simplicity, lack of a need for skin grafting of any donor site, and the single-staged nature of the flap. The primary lobe of the flap was designed over the dorsum of the right middle finger proximal phalanx. This was of a similar size to the defect and at approximately a 30° angle. The secondary lobe was designed over the radial half of the dorsum of the proximal portion of the proximal phalanx of the right index finger (Figure 2). The secondary lobe was slightly narrower than the primary lobe and at a 60° angle from the defect. The flap was raised using a combination of sharp dissection and electrocautery under tourniquet control with care to leave the paratenon over the extensor tendons of the index and middle fingers. The tourniquet was released to confirm adequate perfusion of the flap. The flap was then transposed and sutured with fine non-absorbable sutures. The skin flaps were undermined to permit the primary closure of the secondary defects.

**Figure 3:**
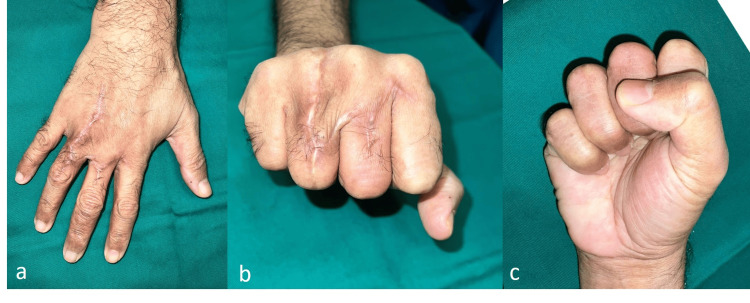
Postoperative appearance: (a) Full finger extension. (b and c) Full finger flexion.

The patient was permitted to commence active range of motion exercises immediately. The flap healed uneventfully. At two months postoperatively, the patient resumed manual labor. The flap healed well and the patient was able to make a full fist and fully extend all fingers (Figure 3).

## Discussion

Reconstruction of dorsal finger defects poses a unique set of challenges. Techniques to cover small defects include traditional rotation or transposition flaps [12], while larger defects may be covered with the reverse dorsal metacarpal artery perforator flap [1] or reverse cross-finger flap [3]. The reverse dorsal metacarpal artery perforator flap is associated with venous congestion, and it may not be feasible on the ulnar side of the hand where there may be absence of dorsal metacarpal artery perforators in the ulnar intermetacarpal spaces [13,14]. In cases where multiple surgeries have been performed, these local perforators may have also been compromised. The reverse cross-finger flap is a two-staged procedure and requires additional skin graft for coverage of the donor site.

The bilobed flap is a double transposition flap that can be used in areas where a single transposition flap does not provide adequate tissue coverage. It recruits skin from adjacent sites with more tissue laxity permitting a tension-free closure [15,16]. It has a history of more than 100 years with the first description being for nasal defect reconstruction [5]. The original description employs a 180° rotation arc and was modified by Zitelli by reducing the rotation arc to around 90° with wide undermining to reduce the pin cushioning effect [6]. For dorsal finger reconstructions, the bilobed flap has been described for small defects after mucous cyst excision [9,17], and for dorsal thumb defects over the proximal phalanx [10].

In Zitelli’s design, the first lobe is the same size as the defect, and the second lobe is undersized by 10% to 15% but slightly longer with a Burow’s triangle included to prevent dog ear formation. Miller and colleagues [18] examined the origination point and rotation arc of the bilobed flap and concluded that it is optimal for each lobe to rotate approximately 45° for a total arc of rotation of 90°. The reason for this is that a more acute angle diminishes the Z-plasty advantage of a double transposition, while a more obtuse angle narrows the flap pedicle. They also found that the angle to the apex of the standing cone should be 30° to avoid puckering at the apex and can decrease tension when the flap is transposed.

We have designed the first lobe to be the same size and the second lobe to be slightly smaller (undersized 20%) to decrease tension at the donor sites. In our patient, the distance from the pivot point of the flap to the distal end of the defect is the same as the distance from the pivot point to the apex to the distal edge of the first lobe and the second lobe. The total arc of rotation was 60° instead of the classical 90° to recruit skin from the dorsum of the adjacent digit distal to the webspace. The tertiary defect was closed first as this pushed the flap toward the primary defect more effectively [18].

This flap does have its limitations, however. As the skin is recruited from the adjacent digits, it can result in finger stiffness and attention should be paid to the range of motion of the adjacent digits, and hand therapy may be required to prevent this or to address it. The extension of the flap across the webspace may result in webspace contracture and a thickening of the web with an appearance similar to that of web creep. Contracture release and Z-plasty may be required to address these complications should they develop.

## Conclusions

This design for a bilobed flap is suitable for soft tissue reconstruction of defects over the dorsum of the metacarpophalangeal joint and the proximal phalanx. It is a simple, reliable, single-staged procedure that provides like-for-like reconstruction and has minimal donor site morbidity.
